# Once-weekly 2.4 mg Semaglutide for Weight Management in Obesity: A Game Changer?

**DOI:** 10.17925/EE.2022.18.1.35

**Published:** 2022-06-15

**Authors:** Ides M Colin, Katherine M Gérard

**Affiliations:** 1. Endocrino-Diabetology Research Unit, Department of Internal Medicine, Centre Hospitalier Régional (CHR) Mons-Hainaut/Groupe Jolimont, Mons, Belgium; 2. Group of Animal Molecular and Cellular Biology, Louvain Institute of Biomolecular Science and Technology (LIBST), Université catholique de Louvain (UCLouvain), Louvain-La-Neuve, Belgium

**Keywords:** Obesity, semaglutide, STEP clinical programme, treatment of obesity, weight-centric approach, weight loss

## Abstract

The treatment of obesity can no longer be reduced to a simplistic view of weight loss. Metabolic adaptation leads to systematic weight regain following weight-loss efforts, and new obesity treatments should therefore aim to induce long-standing double-digit weight loss, and thus improve and even reverse obesity-associated comorbidities such as type 2 diabetes. Until now, only metabolic surgery has been able to achieve such a goal, but this invasive procedure cannot be offered on a large scale. Among the alternatives, lifestyle interventions and drug therapies have often been disappointing. The recent availability of once-weekly subcutaneous 2.4 mg semaglutide (a glucagon-like peptide-1 receptor agonist; Wegovy™ Novo Nordisk A/S, Bagsværd, Denmark) has changed the scene, and semaglutide is considered a ‘game changer’ in the treatment of obesity. The results from the phase III STEP (Semaglutide treatment effect in people with obesity) clinical programme have shown that semaglutide provides clinically meaningful and sustained weight loss in ranges much higher than those achieved with previously available pharmacotherapies. These results led to the approval of semaglutide by regulatory authorities as an adjunct to a reduced-calorie diet and increased physical activity in people with obesity or overweight, with at least one weight-related comorbidity. With data from phase II and III clinical trials showing that newer drugs (i.e. the glucagon-like peptide-1 and gastric inhibitory polypeptide dual receptor agonist tirzepatide and the amylin agonist cagrilintide, either alone or combined) produce a greater sustained weight loss than semaglutide, an upstream ‘weight-centric’ strategy has emerged as a new standard for the treatment of type 2 diabetes.

Obesity – a chronic, relapsing, progressive disease that is defined by a body mass index (BMI) of ≥27.5 kg/m^2^ in Asian populations and ≥30.0 kg/m^2^ in all other populations – is an epidemic that has spread across the world.^1^ Recent data from the World Health Organization (WHO) indicate that 59% of adults and almost one in three children (29% of boys and 27% of girls) in Europe are overweight or living with obesity.^2^ According to the WHO, the prevalence of obesity in the Europe is higher than in any other WHO region except for America, and is among the leading causes of death and disability in this region. Of note, while obesity has been a health issue in high-income countries for many years, the greatest rise in its incidence is now seen in low- and middle-income countries.^3^

Obesity should not be reduced to a simplistic view of ‘too much weight’, but rather be viewed as a driving force behind many health problems. Obesity should be defined as ‘abnormal or excessive fat accumulation that presents a risk to health’.^4^ More than 200 complications are linked to obesity, thereby explaining the increased morbidity and mortality related to the condition and the 4 million obesity-related deaths worldwide in 2015.^5,6^ Obesity (even moderate when the visceral adipose tissue is involved) is associated with an increased risk of developing metabolic syndrome, type 2 diabetes (T2D), cardiovascular disease (CVD) (including hypertension, hyper- or dyslipidaemia, ischaemic heart disease, heart failure, ischaemic stroke), mechanical dysfunction (osteoarthritis), obstructive sleep apnoea syndrome (OSAS), malignancies (e.g. breast cancer, prostate cancer) and mental health issues, including depression, anxiety and eating disorders.^7–11^ Lately, we have even learned that people with obesity are at higher risk of developing more severe disease and complications from COVID-19.^12^ The quality of life (QoL) of people with obesity or overweight is often impaired and their lifespan significantly reduced.^13^ A high BMI is associated with decreased life expectancy of up to 10 years compared with those with healthy BMI and, for every 5 kg/m^2^ BMI increment above the range of 22.5–25.0 kg/m^2^, overall mortality is increased by 30%.^14^ CVD is the leading cause of death in people with obesity, followed by T2D.^6^ In addition to the individual burden, the economic cost associated with obesity should also be noted. The estimated total cost of high BMI to health services worldwide is US$990 billion per year, which represents 13.2% of total healthcare expenditure.^3^

## Pathophysiology

The pathophysiology of obesity is multifactorial and involves social determinants, genetic predisposition and metabolic, behavioural, psychological and environmental factors.^15^ Together, these factors alter the energy balance by modifying satiety and feeding signalling in the brain. Obesity occurs when the energy intake exceeds the total body energy expenditure.^16^ Three areas of the brain, namely the hypothalamus, the mesolimbic area and the prefrontal cortex (executive functioning areas), are directly involved in the control of energy balance.^17–19^ Homeostatic mechanisms include peripheral signals from the gastrointestinal (GI) tract (for food intake information) and from the adipose tissue (for energy use) that are transmitted via the vagus nerve through the central nervous system, where they are processed in the brain stem and hypothalamus. Neural pathways specialized in satiety, hunger and energy expenditure are permanently activated.^17–19^ These pathways have been programmed through millennia of human phylogenetic development so that homeostasis maintains a person's weight at the highest possible level.^19,20^

**Table 1:** Health improvements that are seen with weight loss

Weight loss (%)	Overall health improvements
0–5	Improvement of hypertension and hyperglycaemia
5–10	Prevention of type 2 diabetes, non-alcoholic fatty liver disease, polycystic ovary syndrome Improvement of dyslipidaemia
10–15	Reduction of cardiovascular disease and urinary stress Improvement of non-alcoholic steatohepatitis, obstructive sleep apnoea syndrome, gastro-oesophageal reflux disease, osteoarthritis
>15	Type 2 diabetes remission Improvement and reduction of cardiovascular mortality and heart failure with preserved ejection fraction

This explains why any weight loss immediately triggers compensatory neurohormonal pathways (e.g. involving leptin, ghrelin, peptide YY, gastric inhibitory peptide, slowing of resting metabolism) that increase hunger and decrease energy expenditure – as metabolic adaptation – until a new set-point of energy balance is reached.^19,20^ Thus, even if people with obesity temporarily succeed in losing weight, they immediately face millennia of adaptation leading to weight regain, often to an even greater level from where they started. Given the complex pathophysiology of obesity and the potent counterregulatory neuroendocrine pathways activated to counter weight loss, obesity can no longer be attributed to a lack of willpower. Rather, it should be treated like other chronic cardiometabolic conditions such as T2D, hypertension and hyper- or dyslipidaemia, with treatment aiming to disrupt long-term acquired biochemical circuits.^21,22^ Losing weight and maintaining weight loss over the long term will always be a challenge. Meeting this challenge, both medically and at a societal level, has become unavoidable as the consequences of obesity in terms of health and economic costs become ever clearer.

Reducing obesity improves not just QoL and wellbeing, but also obesity-related comorbidities. Weight loss of 5% is associated with improved blood pressure and glycosylated haemoglobin (HbA1c) levels. Weight loss of 5–10% is associated with reduced intrahepatocellular lipids in non-alcoholic fatty liver disease, reduced triglyceride and non-high-density lipoprotein cholesterol levels, increased high-density lipoprotein cholesterol levels, improved ovulation and prevention of T2D. Weight loss of 10–15% improves physical function in people with osteoarthritis, gastro-oesophageal reflux disease, OSAS, inflammation and fibrosis in non-alcoholic steatohepatitis, and incontinence. More than 15% weight loss may be associated with remission of T2D (which does not mean cure), improvement in heart failure and reduced cardiovascular mortality (*Table 1*).^23^

## Treating obesity: lifestyle measures and metabolic surgery

Although lifestyle interventions, including calorie reduction, daily exercise and behavioural therapy, are considered the cornerstone of treating obesity, it must be recognized that weight loss achieved through these measures is rarely clinically meaningful and often not sustainable over the long term. Clearly, other means, including drug therapies and metabolic surgery (also known as bariatric or metabolic surgery), must be used to promote weight loss. Metabolic surgery is a powerful tool for treating obesity.^24–27^ It can induce weight loss of up to 15–30% of baseline body weight, along with reduced long-term mortality and remission of T2D. One of the major lessons we have learned from metabolic surgery, through double-digit weight loss, is its ability to disrupt the pathophysiological evolution of T2D. The term ‘metabolic’ arose from the findings that Roux-en-Y gastric bypass and sleeve gastrectomy induce deep changes in gut hormone and insulin secretion and sensitivity, independent of weight.^28,29^ A more anorectic hormonal profile helps patients to lose weight and counters increased appetite over the long term. However, metabolic surgery is not a trivial procedure. Several complications can arise in both the short- and long-term postoperative periods. Without long-term medical control, patients may suffer from micronutrient deficiencies, and the risk of weight regain and recurrence of T2D is real.^30,31^

The association between the benefits of metabolic surgery and increased secretion of gut hormones such as glucagon-like peptide (GLP)-1 has led to the development of a class of drugs active in both obesity and T2D. GLP-1 is an incretin hormone (a 30-amino acid peptide hormone) that is continuously released by enteroendocrine cells in the small intestine, but in greater amounts in response to food intake. Its main physiological actions include enhancing glucose-dependent insulin secretion, suppressing postprandial glucagon secretion, slowing gastric emptying and inducing satiety through hypothalamic stimulation.^32^ Long-acting GLP-1 receptor agonists (GLP-1 RAs) have therefore been engineered for the treatment of T2D and obesity.^33–35^

## Treating obesity: drug therapy to date

Until recently, the development of new drugs in the field of obesity has been marked by resounding failures. Some drugs have been withdrawn from the market due to serious side effects, including increased morbidity and mortality.^36–40^ As of June 2021, the US Food and Drug Administration (FDA) had approved only four medications for weight loss: phenterminetopiramate (Qsymia^®^; VIVUS LLC., Campbell, CA, USA), orlistat (Xenical^®^; CHEPLAPHARM Arzneimittel GmbH, Griefswald, Germany), naltrexonebupropion (Contrave^®^; Currax Pharmaceuticals LLC, Brentwood, TN, USA) and the GLP-1 RA liraglutide (Saxenda^®^; Novo Nordisk A/S, Bagsværd, Denmark). Phentermine is also available as monotherapy, but only for short-term (3-month) weight management, while the previously approved agent lorcaserin (Belviq^®^, Belviq XR; both Eisai Inc., Tokyo, Japan) was recalled in early 2020 because of an increased risk of malignancies.^40^ At the same point, the European Medicines Agency (EMA) had approved three drugs: orlistat, naltrexone-bupropion and liraglutide.

The therapeutic performance of these drugs is somewhat disappointing, with 3–5% weight loss in patients treated with orlistat and 5–10% in those receiving naltrexone, liraglutide or phentermine.^36–40^ Liraglutide, which is a GLP-1 RA that was initially developed for the treatment of T2D under the trade name Victoza^®^ (Novo Nordisk A/S, Bagsværd, Denmark; approved for use in T2D in 2009 by the EMA and in 2010 by the FDA), was approved in 2014 for the treatment of obesity up to a maximum dose of 3 mg (Saxenda) in people with a BMI of ≥30 kg/m^2^ or ≥27 kg/m^2^ and obesity-related comorbidities. The approval was based on the SCALE (Satiety and clinical adiposity—liraglutide evidence) phase IIIa programme, in which >3,000 individuals received a daily dose of 3 mg liraglutide.^41^ Those receiving liraglutide lost an average of 8% body weight versus 2.6% with placebo at 56 weeks, thereby reaching the ≥5% difference between active treatment and placebo that is defined by the FDA as the reference for weight-loss drugs.^41^

Once-weekly subcutaneous 2.4 mg semaglutide (Wegovy™; Novo Nordisk A/S, Bagsværd, Denmark) is the newest medication approved for chronic weight management. It is approved as an adjunct to a reduced-calorie diet and increased physical activity in people with obesity or overweight and at least one weight-related comorbidity, including dysglycaemia (prediabetes or T2D), hypertension, dyslipidaemia, OSAS or CVD.^42^ Like liraglutide, semaglutide belongs to the GLP-1 RAs class of drugs. As a long-acting agent, semaglutide reduces energy intake by decreasing appetite and increasing satiety by direct activation of the hypothalamus and hindbrain and indirect activation via the vagus nerve. Compared with liraglutide, semaglutide appears to have better uptake in the brain, which may explain its greater efficacy in weight control and in reducing energy intake (by twice that measured with liraglutide: 35% versus 16%).^33^ The decision to approve this new medication was taken by the EMA in November 2021 (after a submission in December 2020), following a similar decision by the UK Medicines and Healthcare Products Regulatory Agency in September 2021 and by the FDA in June 2021 (again after a submission in December 2020).^43,44^

Lower-dose formulations of semaglutide (0.25, 0.5 and 1.0 mg [Ozempic^®^; Novo Nordisk A/S, Bagsværd, Denmark]) had previously been approved in Europe and the USA for the treatment of T2D.^45,46^ The SUSTAIN (Semaglutide unabated sustainability in treatment of type 2 diabetes) clinical development programme (with >8,000 people with T2D) demonstrated, in addition to T2D improvement, that weekly semaglutide induced significant weight loss.^47^ For instance, in the placebo-controlled clinical trial SUSTAIN-1, around 40% of participants on semaglutide achieved a weight loss of 5% or more. In November 2021, the EMA extended the dose of once-weekly subcutaneous semaglutide up to 2 mg for the treatment of T2D.^46^ Of note, an oral tablet form of semaglutide (Rybelsus^®^ [Novo Nordisk A/S, Bagsværd, Denmark] 3, 7 and 14 mg) had previously been approved in the USA in September 2019 and in Europe in January 2020 for the treatment of T2D.^48,49^ Due to the need for these approvals to be achieved, this drug has only recently become available on the market.

## The STEP phase III clinical programme

The decision of regulatory authorities to approve once-weekly subcutaneous 2.4 mg semaglutide as an anti-obesity medication was based on data from four trials of the STEP (Semaglutide treatment effect in people with obesity) phase IIIa clinical programme, which involved >4,500 people.^42,50^ The STEP clinical programme was a 68-week, randomized, double-blind, placebo-controlled, two-armed (three-armed in STEP 2; see below), parallel-group, multicentre, multinational clinical programme, with 7 weeks of follow-up without treatment for safety assessments. The programme compared 2.4 mg semaglutide with placebo, as an adjunct to lifestyle interventions (500 kcal/day deficit relative to the estimated total energy expenditure, plus 150 minutes/week of physical activity) (except for STEP 3; see below), in people with obesity or overweight (and T2D in STEP 2). The main primary endpoints were percentage change in body weight and the proportion of participants with ≥5% weight loss from baseline (i.e. at randomization; week 20 in STEP 4) to week 68, without weight regain. The safety and tolerability profiles were also thoroughly investigated.

Participants were aged ≥18 years and had a history of at least one self-reported unsuccessful dietary effort to lose body weight. Except in STEP 2, adults in the STEP clinical programme had BMI ≥30 kg/m^2^ or ≥27 kg/m^2^ with the presence of weight-related complications (treated or untreated): dyslipidaemia, OSAS, hypertension or CVD. Participants in STEP 2 were also required to be diagnosed with T2D (HbA1c 7–10% [53–86 mmol/mol]) ≥180 days prior to screening. In all of the clinical trials, semaglutide was administered subcutaneously using a prefilled pen (4 mm, 32-gauge needle) at an initial dose of 0.25 mg/week for the first 4 weeks, followed by a 12-week dose escalation (0.5, 1.0 and 1.7 mg/week each for 4 weeks) up to 2.4 mg/week by the end of week 16, depending on individual tolerance (with 1.7 mg once weekly permitted for those who were not able to reach the 2.4 mg dose, but with at least one attempt to re-escalate to the full 2.4 mg dose). Participants were treated for 52 weeks (48 weeks in STEP 4) on the maintenance dose.

**Figure 1:**
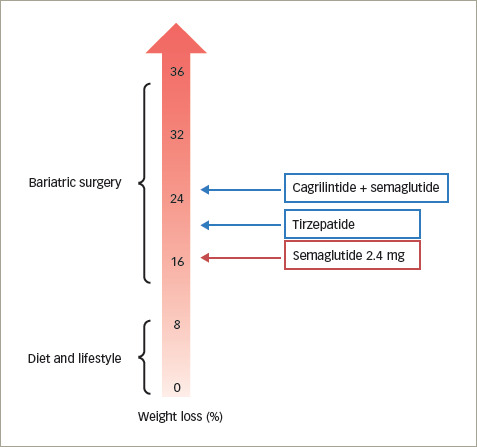
Treatments for obesity and their impact on weight loss

Briefly, STEP 1 (n=1,961; STEP 1: Research study investigating how well semaglutide works in people suffering from overweight or obesity (STEP 1); ClinicalTrials.gov identifier: NCT03548935) looked at weight management in a general population of people with obesity or overweight;^51^ STEP 2 (n=1,210; Research study investigating how well semaglutide works in people with type 2 diabetes suffering from overweight or obesity (STEP 2); ClinicalTrials.gov identifier: NCT03552757) was about weight management in people who also had T2D;^52^ STEP 3 (n=611; Research study to look at how well semaglutide is at lowering weight when taken together with an intensive lifestyle program (STEP 3); ClinicalTrials.gov identifier: NCT03611582) added intensive behavioural therapy to the protocol;^53^ and STEP 4 (n=803; Research study investigating how well semaglutide works in people suffering from overweight or obesity (STEP 4); ClinicalTrials.gov identifier: NCT03548987) studied sustained weight management over 48 weeks in people who received 2.4 mg semaglutide during an initial 20-week runin period.^54^ Of note, a long-term weight maintenance trial (STEP 5; n=304; Two-year research study investigating how well semaglutide works in people suffering from overweight or obesity [STEP 5]; ClinicalTrials.gov identifier: NCT03693430) recently came to an end, with weight loss assessed over a 2-year period.^55^ Compared with participants on placebo, those who received subcutaneous once-weekly 2.4 mg semaglutide had an average weight loss of up to 16%, which comes close – although not yet at the same level – to the performance of metabolic surgery (*Figure 1*).^42^ This is the first time that double-digit weight loss has been achieved by pharmacological means. The STEP clinical programme is therefore considered by many experts to be a breakthrough in the field of obesity. The safety profile showed that mild-to-moderate GI symptoms (nausea and vomiting) were predominant in people receiving semaglutide. Semaglutide-induced hypoglycaemia was not an issue, making this drug safe when administered to people without T2D.

### STEP 1

STEP 1 enrolled 1,961 adults with obesity or overweight, with a mean age of 47 years; three-quarters were women.^51^ Participants came from 129 sites in 16 countries across Asia, Europe, North America and South America. Most were White (76%), followed by Asian (13%), Black/African American (6%) and ‘other’ (5%). Participants were randomly assigned in a 2:1 manner to receive 2.4 mg semaglutide (n=1,306) or placebo (n=655) once weekly for 68 weeks, in addition to lifestyle intervention. T2D was an exclusion criterion, but 40–45% of the cohort had prediabetes (defined in accordance with American Diabetes Association (ADA) criteria).^56^ A subpopulation of 140 participants had their body composition assessed by dual-energy X-ray (DXA) absorptiometry to test whether any weight loss was primarily caused by a reduction in fat mass.

At week 68, individuals in the semaglutide group had greater mean weight loss from baseline than those in the placebo group (14.9% versus 2.4%; estimated treatment difference -12.4 percentage points; 95% confidence interval [CI] -13.4 to -11.5; p<0.001).^51^ The change in body weight from baseline to week 68 was -15.3 versus -2.6 kg, respectively (estimated treatment difference -12.7 kg; 95% CI -13.7 to -11.7). Overall, 86.4%, 69.1% and 50.5% of participants receiving semaglutide had weight loss of >5%, >10% and >15%, respectively, compared with 31.5%, 12.0% and 4.9% of those on placebo (p<0.001 for all comparisons). Furthermore, 32.0% of participants in the semaglutide group lost ≥20% of their initial body weight, which is close to the results seen with surgical procedures. Participants in the semaglutide group also had greater improvements in waist circumference, C-reactive protein plasma levels and fasting lipids than those on placebo. Physical function scores (measured using the Short-Form 36 Health Survey and Impact of Weight on Quality of Life, Lite Clinical Trials [IWQOL-Lite-CT] questionnaires) were also greatly improved, which is of particular note considering the impact of obesity on physical and mental health-related QoL.^57,58^ In the DXA subpopulation, total fat mass and regional visceral fat mass were reduced from baseline with semaglutide.

Adverse effects were in line with those expected with GLP-1 RAs, with mild-to-moderate GI events. There were also more gallbladder disorders, mostly cholelithiasis, in the semaglutide group (2.6% versus 1.2% in the placebo group), likely associated with pronounced weight loss. More participants in the semaglutide group than in the placebo group discontinued treatment owing to GI events (4.5% versus 0.8%).^51^

### STEP 2

STEP 2 was a double-blind, double-dummy superiority study that enrolled 1,210 adults (mean age 55 years; 50.9% women; 62.1% White, 26.2% Asian, 11.6% Hispanic, 8.3% Black/African American, 4% other) with obesity or overweight (mean BMI 35.7 kg/m^2^) and T2D (mean HbA1c 8.1%; diabetes duration 8 years; low cardiovascular risk profile) from 149 sites in 12 countries across Europe, North America, South America, the Middle East, South Africa and Asia.^52^ Prior to entering the study, T2D was managed with diet and exercise alone or with treatments including stable doses of up to three oral glucose-lowering agents (metformin, sulfonylureas, sodium-glucose co-transporter-2 inhibitors or thiazolidinediones) for ≥90 days. Participants were randomly assigned in 1:1:1 ratio to receive 2.4 mg semaglutide (n=404), 1.0 mg semaglutide (n=403) or placebo (n=403) once weekly for 68 weeks, plus the same lifestyle intervention as in STEP 1.

Change in mean body weight from baseline to week 68 was –9.6% in the 2.4 mg semaglutide group, -7.0% in the 1.0 mg semaglutide group and -3.4% in the placebo group. The estimated treatment difference for 2.4 mg semaglutide versus placebo was -6.2 percentage points (95% CI -7.3 to -5.2; p<0.0001). Weight loss of ≥5% was achieved in 68.8% of participants in the 2.4 mg semaglutide group versus 57.1% of those in the 1.0 mg semaglutide group and 28.5% of those in the placebo group (2.4 mg semaglutide versus placebo: odds ratio 4.88, 95% CI 3.58 to 6.64; p<0.0001). The corresponding rates for ≥10% weight loss were 45.6%, 28.7% and 8.2%, and for ≥15% weight loss were 25.8%, 13.7% and 3.2%. More than 25% of participants on active treatment lost >15% of body weight. The mean HbA1c level decreased by 1.6% (17.5 mmol/mol) and 1.5% (15.9 mmol/mol) in the 2.4 and 1.0 mg semaglutide groups, respectively, and by 0.4% (4.1 mmol/mol) in the placebo group. Overall, 28.6% of participants in the 2.4 mg semaglutide group were able to reduce their glucose-lowering medications, versus 25.1% of participants on the 1.0 mg dose and 7.1% of those on placebo. As in STEP 1, once-weekly 2.4 mg semaglutide was associated with improvement in cardiometabolic risk factors (systolic blood pressure, C-reactive protein plasma levels and triglycerides) and physical functioning scores. GI adverse events (mostly mild to moderate) were reported by 64% of participants in the 2.4 mg semaglutide group, 58% in the 1.0 mg semaglutide group and 34% in the placebo group.^52^

### STEP 3

The STEP 3 clinical trial was conducted at 41 sites in the USA only, and recruited 611 adults (mean age 46 years; 81% women; 76% White and 19% Black/African American) with obesity or overweight, but without T2D.^53^ Participants were randomly assigned in a 2:1 manner to receive either 2.4 mg semaglutide (n=407) or placebo (n=204) for 68 weeks. The treatment was administered as an adjunct to a low-calorie diet for the first 8 weeks, followed by 60 weeks of intensive behavioural therapy including a hypocaloric diet and increased physical activity. The initial 8-week low-calorie diet comprised a 1,000–1,200 kcal/day mealreplacement diet (in order to induce strong initial weight loss), and was followed by a 1,200–1,800 kcal/day hypocaloric diet of conventional food over the remaining 60 weeks. Participants were also asked to perform ≥100 minutes/week of physical activity, which increased by 25 minutes every 4 weeks to finally reach 200 minutes/week by month 6. They received 30 individualized 20- to 30-minute intensive behavioural therapy sessions with a registered dietitian.

**Figure 2:**
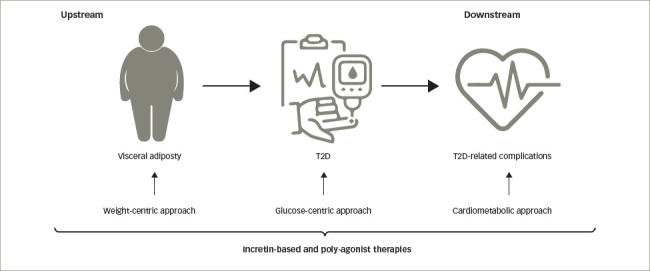
An upstream weight-centric approach versus more downstream, glucose-centric and cardiometabolic approaches. Incretin-based therapies are already active at the most upstream step

*T2D = type 2 diabetes mellitus.*

At week 68, mean body weight had decreased from baseline by 16.0% in the semaglutide group versus 5.7% in the placebo group – a mean difference of -10.3 percentage points (p<0.001). A higher proportion of participants on semaglutide had weight loss of ≥5%, ≥10%, ≥15% and ≥20% of their initial weight versus those on placebo (86.6% versus 47.6%; 75.3% versus 27.0%; 55.8% versus 13.2%; and 35.7% versus 3.7%, respectively; all p<0.001). The weight loss achieved with semaglutide in STEP 3 was therefore similar to that in STEP 1, which involved less-intensive lifestyle intervention. Thus, while lifestyle interventions should always be included as background therapy prior to and during semaglutide administration, it appears that moderate (versus intensive) behavioural therapy without an initial low-calorie diet is enough to achieve significant weight loss.

From baseline to week 68, the proportion of participants with prediabetes, defined using the ADA criteria, dropped from 48% to 7% in the semaglutide group and from 53% to 26% in the placebo group. Those on semaglutide also had greater improvements in lipids, HbA1c, fasting plasma glucose, insulin, waist circumference and systolic blood pressure. As noticed in other trials, GI adverse events were mild to moderate and occurred in 82.8% of people in the semaglutide group and 63.2% of the placebo group; 3.4% of participants on semaglutide discontinued the treatment because of these events.^53^

### STEP 4

STEP 4 can be considered as a multicentre, phase IIIa ‘withdrawal’ trial designed to study weight changes after switching from semaglutide to placebo.^54^ A total of 902 participants (79% women; 84% White, 13% Black/Afro-American, 2% Asian, 1% other; 7.8% Hispanic/Latino ethnicity; mean age 46.0 years) with obesity or overweight (mean BMI 38.4 kg/m^2^), at least one weight-related comorbidity and without T2D were recruited from 73 sites in 10 countries. Participants were started on treatment with once-weekly semaglutide in incremental doses (an initial dose of 0.25 mg/week for the first 4 weeks, followed by a 12-week dose escalation of 0.5, 1.0 and 1.7 mg/week each for 4 weeks and then up to 2.4 mg/week by the end of week 16) to reach a plateau of 2.4 mg by the end of a 20-week, open-label run-in phase.

By the end of the run-in phase (16 weeks of dose escalation and 4 weeks of maintenance dose), 803 participants – who already had a mean loss of 10.6% of their baseline weight – were randomized in a 2:1 manner to either continue 2.4 mg semaglutide or receive placebo (plus lifestyle intervention in both groups) for an additional 48 weeks. The primary endpoint was percentage change in body weight from week 20 to week 68. By the end of the randomized period (week 68), participants on semaglutide had lost an additional 7.9% of their weight from the week 20 timepoint (with a plateau reached at week 60–68), while those switched to placebo had gained 6.9% (difference 14.8%, 95% CI -16.0 to -13.5; p<0.001). Participants in the semaglutide group also had significant improvements in waist circumference, blood glucose, systolic blood pressure and Short-Form 36 physical functioning score. By the end of the 68-week period, participants kept on semaglutide had lost 17.4% of their initial baseline weight – with ≥15% weight loss in 64% of participants and ≥20% weight loss in 40% (which is again within the range of results obtained by sleeve gastrectomy) – versus 5.0% in those switched to placebo. Those who switched to placebo at week 20 gradually regained weight.

The adverse event profile was similar to that seen in other studies with GLP-1 RAs. The most common adverse events again concerned the GI tract, with just over 2% of participants in each arm discontinuing treatment in the randomized period because of adverse events.^54^

### STEP 8

The results of the STEP 8 clinical trial (Research study to investigate how well semaglutide works compared to liraglutide in people living with overweight or obesity [STEP 8]; ClinicalTrials.gov identifier: NCT04074161) were published in January of this year.^59^ The regulatory authorities did not take this trial into account when making their decision regarding the licensing of semaglutide. STEP 8 was a randomized, open-label, 68-week phase IIIb trial, with 338 adults (mean age 49.0 years; 78.4% women) with overweight or obesity and at least one weight-related comorbidity, but without diabetes, recruited at 19 sites in the USA. The objective of this trial was to compare the efficacy and adverse event profiles of once-weekly subcutaneous 2.4 mg semaglutide (n=126) versus once-daily subcutaneous 3.0 mg liraglutide (n=127) or matched injected placebo (n=85) (all with counselling on diet and physical activity). In contrast to semaglutide, for which the target dose was reached after 16 weeks, the target liraglutide dose was reached after only 4 weeks.

The mean change in body weight from baseline to week 68 (the primary outcome) was -15.8% with semaglutide versus -6.4% with liraglutide (estimated treatment difference -9.4%; 95% CI -12 to -6.8; p<0.001). This corresponded to an average decrease of 15.3 and 6.8 kg, respectively, from starting body weights of 102.5 kg and 103.7 kg. Higher proportions of participants on semaglutide versus placebo had weight loss of ≥10%, ≥15% and ≥20% of their initial weight (70.9% versus 25.6%; 55.6% versus 12.0%; and 38.5% versus 6.0%, respectively; all p<0.001). People receiving weekly 2.4 mg semaglutide versus daily 3.0 mg liraglutide had significantly greater reductions in waist circumference and lipid, HbA1c and C-reactive protein plasma levels. Not surprisingly, GI adverse events were again most common and were reported by 84.1% of participants receiving semaglutide, 82.7% of those on liraglutide and 55.3% on placebo. They were mostly transient and mild to moderate in severity. Fewer participants stopped treatment due to adverse events with semaglutide than liraglutide (3.2% versus 12.6%). Based on these results, it clearly appears that 2.4 mg semaglutide produces greater long-term weight loss than once-daily injected 3.0 mg liraglutide in adults with overweight or obesity, but without diabetes.

### Other STEP trials

Other STEP trials are foreseen in the future. Although completed, the results of the STEP 5 clinical trial have not yet been published.^55^ This trial is similar to STEP 1, as it aims to test the durability of weight loss with 2.4 mg semaglutide versus placebo, but over a period of 2 years in 304 participants. Preliminary reports indicate that, in addition to lifestyle intervention, weekly injection of 2.4 mg semaglutide led to weight loss from baseline to week 104 of 15.2% in the active group versus 2.6% in the placebo group (p<0.0001), with 77% and 34% of participants, respectively, losing ≥5% of their body weight (p<0.0001).^60^

STEP 6 (STEP 6: Research study investigating how well semaglutide works in people living with overweight or obesity; ClinicalTrials.gov identifier: NCT03811574) was a randomized, double-blind, double-dummy, placebo-controlled, phase IIIa superiority trial.^61^ The trial was similar to STEP 1, but was performed at 28 outpatient clinics in Japan and South Korea. The results of STEP 6 were published in February 2022.^61^ A total of 401 participants from Japan (with diabetes) and South Korea were randomized to receive 1.7 mg semaglutide (n=101), 2.4 mg semaglutide (n=199) or placebo (n=101) once weekly for 68 weeks. STEP 6 showed that, when administered to adults from East Asia with overweight or obesity, with or without T2D, once-weekly 1.7 and 2.4 mg semaglutide induced superior and clinically meaningful reductions in body weight compared with placebo.^61^ At week 68, individuals in the semaglutide versus placebo groups had greater mean weight loss (2.4 mg semaglutide versus placebo: estimated treatment difference -11.1 percentage points, 95% CI -12.9 to -9.2; 1.7 mg semaglutide versus placebo: estimated treatment difference -7.5%, 95% CI -9.6 to -5.4; both p<0.0001), and a greater reduction in the abdominal visceral fat area. Overall, 86% of participants in the 2.4 mg semaglutide group, 82% in the 1.7 mg semaglutide group and 79% in the placebo group reported adverse events. GI disorders, which were mostly mild to moderate, were the most frequent. Adverse events leading to discontinuation occurred in 3% of participants in the semaglutide groups and 1% of those in the placebo group.

STEP 7, which is active but not yet recruiting (ClinicalTrials.gov identifier: NCT04251156), aims to enrol 375 people (with or without T2D) across China, Hong Kong, South Korea and Brazil.^62^ Participants will receive 2.4 mg semaglutide versus placebo for 44 weeks. STEP 10 (ClinicalTrials.gov identifier: NCT05040971) aims to study the ability of once-weekly 2.4 mg semaglutide, administered for 52 weeks, to reverse prediabetes in 201 participants from Canada, Finland and Spain.^63^ STEP TEENS (ClinicalTrials.gov identifier: NCT04102189) will study the efficacy and safety of semaglutide in adolescents.^64^

### SELECT

Finally, the SELECT (Semaglutide effects on heart disease and stroke in people with overweight or obesity; ClinicalTrials.gov identifier: NCT03574597) trial is a randomized, double-blind, parallel-group, placebo-controlled trial comparing semaglutide with placebo as an adjunct to standard of care for preventing major adverse cardiovascular events in people (estimated enrolment: 17,500 participants) with established CVD and overweight or obesity.^65^ The results are expected next year.

## Discussion

Treating obesity is not just about weight loss, but also about improving or reversing obesity-related risk factors and health issues. With the publication of several clinical trials on metabolic surgery, obesity began to appear as a clinically manageable and even reversible disease.^25–27^ We also learned that double-digit weight loss (of around 15%) after metabolic surgery could even result in T2D remission.^25–27^ After the results of the first trials from the STEP clinical programme were published, it appeared that such magnitude of weight loss can also be achieved by pharmacological means.^51–54^ Once-weekly 2.4 mg semaglutide has been reported to induce 10–18% weight loss and to significantly improve health, prevent T2D and bring about disease remission (although this last claim has still to be definitively proved). The magnitude of weight loss is much higher (actually, double the weight-loss efficacy) than the 5–10% weight loss achieved with previous behavioural and pharmacological approaches. Therefore, once-weekly 2.4 mg semaglutide is narrowing the gap between pharmacotherapy and metabolic surgery (*Figure 1*). It should be kept in mind that the effects of once-weekly 2.4 mg semaglutide have been evaluated over a period of up to 2 years (STEP 5), compared with metabolic surgery, which has been evaluated over periods sometimes exceeding a decade. Nonetheless, this drug may be considered a less invasive alternative to metabolic surgery in people who do not meet the eligibility criteria for metabolic surgery, do not want to undergo surgery or do not succeed in maintaining long-term weight loss with surgery.^42,50^

With such an impact on weight, the neurohormonal mechanisms driving metabolic adaptation in response to weight loss should be activated with semaglutide treatment. However, 2.4 mg semaglutide is able to disrupt this pathway, as shown by the long-term durability of weight loss.^60^ By doing so, semaglutide solves the second biggest concern associated with weight loss (besides the magnitude of effect) – namely, weight regain. By reversing metabolic abnormalities, semaglutide treatment improves metabolic health function. This has been consistently observed across the STEP clinical programme, with participants experiencing improved QoL in terms of energy gain, less pain, increased movement and feeling better in everyday life.^54,60^

Obesity is a chronic lifelong disease, and the sustained weight loss observed over 2 years in STEP 5 is encouraging. However, further clinical trials and clinical practice with 2.4 mg semaglutide over the long term are needed to verify whether semaglutide can maintain its benefits over the very long term. STEP 4 showed that the weight-loss effect is maintained as long as semaglutide is administered, and stops immediately after discontinuation.^54^ As a chronic disease, it can be argued that obesity should be treated as any other chronic metabolic disorder, with life-long therapy required.^21,22^

Semaglutide is expensive and unaffordable for many people. Economic issues will be raised when its reimbursement is discussed by health authorities. However, as far as economic issues are concerned, metabolic surgery and its long-term follow-up are also very expensive. A cost-effectiveness analysis should therefore be carried out. Any economic discussion should take into account the substantial cost savings that will result from the improvement, prevention and potential remission of obesity-associated diseases, such as T2D, other cardiometabolic disorders and mental health-related problems. Based on the example of statins over the last 30 years, and with the proviso that the evidence regarding the benefits of semaglutide continues to accumulate in the years to come, one may hope that this agent will be used and reimbursed in both primary and secondary prevention and intervention. The development of generic versions of the molecule will make the discussion much easier, but this is far in the future.

One important piece of evidence will be provided by the ongoing SELECT trial, which will study cardiovascular outcome data (e.g. three-point major adverse cardiovascular events) after 2.5–5 years.^65^ These results will be of crucial importance for the future of semaglutide.

As the effects of GLP-1 RAs are glucose dependent, hypoglycaemia is not an issue in people treated with semaglutide. Indeed, the STEP programme has determined that the risk of hypoglycaemia is low. The most frequent adverse events involve the GI system, with nausea, vomiting and diarrhoea being the most frequently reported. These adverse events are rarely severe and tend to be transient. Because semaglutide is gradually uptitrated, GI side effects should be easily contained. Reducing portions sizes and avoiding fatty foods may also help.^42,51–54,59–61,66^ These GI side effects, along with slow gastric emptying, cannot, however, be considered responsible for the drug's effect on weight loss.^66^ Like the other GLP-1 RAs, semaglutide has a black box warning for medullary thyroid carcinoma (although thyroid cancers have not been reported in human trials) and should not be used in patients with a personal or family history of medullary carcinoma or multiple endocrine neoplasia syndrome. There are also no concerns about an increased risk of other cancers. Other serious adverse effects include pancreatitis, increased pancreatic lipases and gallbladder diseases (cholelithiasis and cholecystitis). Treatment with GLP-1 RAs is not recommended in people with a medical history of pancreatitis. Of note, and unlike previous anti-obesity drugs, there are no psychological or psychiatric side effects associated with semaglutide. Compared with previous clinical trials with GLP-1 RAs, no additional safety signals (e.g. retinopathy) have emerged from the STEP clinical programme.^42,50–54,59–61,66^ Globally, patients treated with 2.4 mg semaglutide in the STEP clinical programme have discontinued the treatment due to adverse events at a higher rate (7%) than those on placebo (3.1%), primarily due to GI adverse events (4.5% versus 0.8%).^42,51–54,59–61,66^

Following on from the theory that molecules acting on two or more different receptors would be expected to generate more weight loss than a single receptor agonist, new molecules have been engineered. Among them, tirzepatide (formerly LY3298176) is a novel, once-weekly, injectable, dual glucose-dependent insulinotropic polypeptide (GIP) and GLP-1 RA that integrates GIP and GLP-1 actions into a single molecule.^67^ In a recent open-label phase III clinical trial, three doses of once-weekly tirzepatide induced additional weight loss from baseline to 40 weeks versus once-weekly 1 mg semaglutide in people with T2D.^68^ After 40 weeks of treatment, weight loss of ≥15% was observed in 15–40% of participants receiving tirzepatide versus 9% of those receiving semaglutide.^68^ Of note, doses of semaglutide higher than 1 mg were not compared with tirzepatide.

Another weight-loss drug in the pipeline is the long-acting amylin agonist cagrilintide (AM833). Amylin is a peptide that is co-secreted with insulin and C-peptide from pancreatic β-cells and has important appetite-regulatory effects via central effects on satiety pathways.^69^ A randomized, placebo-controlled, multiple-ascending dose, phase Ib trial (A research study of how NNC0174-0833 taken with semaglutide works in people who are overweight or obese; ClinicalTrials.gov identifier: NCT03600480) recently showed that the combination of once-weekly 2.4 mg semaglutide plus once-weekly 2.4 mg cagrilintide resulted in a 17.1% mean weight loss at week 20 of treatment, compared with 9.8% with semaglutide alone.^70^ Based on these very encouraging preliminary results, we can expect to see weight loss in the range of 20–30% – similar to that seen with metabolic surgery – in the next phase III clinical trial programme.

## Conclusion

The phase III STEP clinical programme has shown that 2.4 mg semaglutide provides clinically meaningful and durable weight loss beyond that achieved with other currently available agents for obesity. It also improves the health and QoL of people with obesity or overweight (with or without T2D).

Double-digit weight loss can be accompanied by T2D remission. As the goal of double-digit weight loss is now achievable with new anti-obesity drugs, the time has come to update how T2D should be treated and to shift the strategy from a glucose-centric approach to a more weight-oriented view, as recently suggested by Lingvay et al.^21^ More than 90% of people with T2D are overweight or have obesity, and >20% of people with obesity have T2D. There is a clear continuous pathophysiological pathway between central adiposity and T2D (*Figure 2*). It therefore makes sense to treat T2D upstream in the pathophysiological continuum. By doing so, a unique treatment (i.e. GLP-1 RAs) will be active from early-onset obesity, to T2D, and to late-onset T2D-related comorbidities. Once-weekly 2.4 mg semaglutide may become essential in the treatment of people with obesity or overweight with or without T2D, because it directly addresses the pathophysiological link between obesity and T2D. It can improve (or prevent) T2D-associated complications, as well as the many physical and mental health problems that are associated with obesity.

To answer the question raised in the title of this review: yes, once-weekly 2.4 mg semaglutide is a ‘game changer’ in the treatment of obesity. However, in view of the newest anti-obesity drugs currently under investigation with even greater potency than once-weekly 2.4 mg semaglutide, it may be that, in the field of obesity, the best is yet to come – and the first ‘STEPS’ will surely be followed by others…
